# Epigenetic assimilation in the aging human brain

**DOI:** 10.1186/s13059-016-0946-8

**Published:** 2016-04-28

**Authors:** Gabriel Oh, Sasha Ebrahimi, Sun-Chong Wang, Rene Cortese, Zachary A. Kaminsky, Irving I. Gottesman, James R. Burke, Brenda L. Plassman, Art Petronis

**Affiliations:** Krembil Family Epigenetics Laboratory, Campbell Family Mental Health Research Institute, Centre for Addiction and Mental Health, 250 College St. R130, Toronto, Ontario M5T 1R8 Canada; Institute of Systems Biology and Bioinformatics, National Central University, Chungli, 320 Taiwan; Department of Pediatrics, University of Chicago, Chicago, Illinois 60637 USA; Department of Psychiatry and Behavioral Sciences, School of Medicine, Johns Hopkins University, Baltimore, Maryland 21287 USA; Departments of Psychology and Psychiatry, University of Minnesota, Minneapolis, Minnesota 55455 USA; Duke University Medical Center, Duke University, Box 2900, Durham, North Carolina 27701 USA; Duke University Medical Center, Duke University, Box 41, Durham, North Carolina 27701 USA

**Keywords:** Epigenetics, Aging, Alzheimer’s disease, DNA methylation, Transcriptome, Genomic organization, Dedifferentiation, Epigenetic drift

## Abstract

**Background:**

Epigenetic drift progressively increases variation in DNA modification profiles of aging cells, but the finale of such divergence remains elusive. In this study, we explored the dynamics of DNA modification and transcription in the later stages of human life.

**Results:**

We find that brain tissues of older individuals (>75 years) become more similar to each other, both epigenetically and transcriptionally, compared with younger individuals. Inter-individual epigenetic assimilation is concurrent with increasing similarity between the cerebral cortex and the cerebellum, which points to potential brain cell dedifferentiation. DNA modification analysis of twins affected with Alzheimer’s disease reveals a potential for accelerated epigenetic assimilation in neurodegenerative disease. We also observe loss of boundaries and merging of neighboring DNA modification and transcriptomic domains over time.

**Conclusions:**

Age-dependent epigenetic divergence, paradoxically, changes to convergence in the later stages of life. The newly described phenomena of epigenetic assimilation and tissue dedifferentiation may help us better understand the molecular mechanisms of aging and the origins of diseases for which age is a risk factor.

**Electronic supplementary material:**

The online version of this article (doi:10.1186/s13059-016-0946-8) contains supplementary material, which is available to authorized users.

## Background

Epigenetic factors regulate DNA sequences, organize them within the nucleus, and contribute to phenotypic variation of normal traits and disease susceptibility [1]. In comparison to the genetic code, epigenetic modifications exhibit a much higher degree of variability, which applies to different individuals, different tissues within an individual, and even the cells within a given tissue. Epigenetic variation is present from the earliest stages of development all the way to old age. Germ cells produced by the same person exhibit a high degree of epigenetic variability [2], some of which may survive post-zygotic epigenomic reprogramming [3]. Throughout embryogenesis and development, cellular epigenomes undergo major re-arrangements: from differentiation of pluripotent stem cells, to lineage-restricted stem cells, and ultimately into somatic cells [4, 5]. For the most part, epigenetic patterns are then “locked” and transmitted through mitosis into subsequent daughter cells [6, 7]. Interestingly, epigenetic changes continue to accrue, although much more gradually, as exogenous (i.e., environmental) [8, 9] and endogenous (i.e., stochastic) [10, 11] factors influence the epigenomes of somatic cells. One of the best understood modes of epigenetic regulation is DNA methylation, which involves the covalent addition of a methyl group to a cytosine, preferentially to a CpG dinucleotide. This is a heritable and reversible process with error rates of 10^−4^–10^−5^ per mitosis in cell cultures [12, 13], which is several orders of magnitude higher than somatic DNA mutation rates [14]. Additionally, de novo DNA methylation has also been estimated at 3–5 % per mitosis [15]. Together with exogenous factors, this yields an imperfectly maintained mechanism that accumulates epigenetic changes over time, resulting in a process referred to as epigenetic drift [16].

Monozygotic (MZ) twins—two individuals perfectly matched for age, sex, and genotype—offer a unique opportunity to evaluate epigenetic drift. Twin studies have consistently identified epigenetic changes in individual genes and whole genomes during development and aging [17–20]. Increases in discordance can be as high as 8–16 % per decade at selected loci, observed both longitudinally and cross-sectionally [17]. Cells apply restraints to the stochastic nature of epigenetic drift, which results in a significantly lower degree of epigenetic variation at CpG islands compared with the surrounding regions [19–21]. This is likely due to the fact that the regulatory and coding parts of the genome are under much more stringent control than intergenic and non-coding DNA regions [22]. Similarly, epigenetic drift has been observed more frequently in parts of the human interactome that display lower connectivity and centrality [23]. From an evolutionary perspective, some degree of epigenetic variability may be advantageous; such fluctuations would yield higher phenotypic variability and increase fitness in changing environments [24, 25].

While age-dependent epigenetic variation can increase significantly, changes in the group mean values of DNA methylation can be subtle. For example, a study found that older twins (>74 years) showed a significantly higher standard deviation in their measure of global DNA methylation compared with younger twins (<30 years old, 1.5-fold; *p* = 2.3 × 10^−5^), although absolute mean difference was only 0.4 % (*p* = 0.03) between the two groups [17]. In another study, global analysis of DNA methylation patterns found a gradual depletion of modified cytosines in mammalian cells with age [26]. While these findings have been validated in other studies [27, 28], more subtleties have also emerged. Age-dependent changes in DNA methylation appear to drift in both directions; methylation tends to decrease in repetitive elements [17, 27] but increase in CpG islands of many key developmental genes [29–31].

There is experimental evidence suggesting that epigenetic fluctuations may stop diverging in very old individuals. In twin studies, variation in global genome methylation increased gradually until 75 years of age but showed a decreasing trend in the oldest twin group (76–88 years) [17]. Likewise, methylation patterns of distant CpGs (1–5 kb) become more similar in older individuals (Figure S6 of [32]). In addition, it was shown that boundaries of topologically associated domains start degrading in senescent cells [33], which is also consistent with a loss of epigenetic complexity.

Our study is dedicated to the analysis of this intriguing, but yet unexplored, phenomenon of putative epigenetic convergence in aging cells and organisms. Since the techniques used for epigenomic DNA studies did not differentiate methylated cytosines from the more recently discovered hydroxymethylated, carboxylated, and formylated cytosines, a general term, DNA modification, will be used in this article. In this regard, we have re-analyzed publically available DNA modification and transcriptomic datasets with subjects across various ages. We have also performed DNA modification profiling of post-mortem brain tissues and a separate set of buccal epithelium samples, from MZ and DZ twins discordant for diagnosis or differing in age of onset of Alzheimer’s disease, to investigate the impact of neurodegenerative disease on epigenetic assimilation.

## Results

### The dynamics of DNA modification and transcription in aging brain

We examined publically available datasets of DNA modification and the transcriptomes in the cerebral cortex and the cerebellum from the North American Brain Expression Consortium: DNA Methylation and the North American Brain Expression Consortium and UK Human Brain Expression Database datasets (Additional file 1: Table S1).

First, we looked for the presence of age-dependent epigenetic drift using the bisulfite conversion-based Illumina 27 K microarray dataset. We tested to see if DNA modification variance increases with age by examining age-dependent heteroscedasticity, which refers to a subset of samples exhibiting different degrees of variability [34]. About 30 % of probes exhibited significant changes in variance with age in both the cerebral cortex and the cerebellum (2708 and 3064 of 10,630 normalized CpG probes, respectively; Harrison–McCabe test, false discovery rate (FDR) q < 0.05). Approximately 90 % of the heteroscedastic loci showed increasing variance with age (2399 of 2708 probes in the cortex; 2822 of 3064 probes in the cerebellum), consistent with previous studies of epigenetic drift showing diverging DNA modification patterns with age.

Multiple pair-wise intraclass correlations (ICC) were used as an estimate of age-dependent epigenetic assimilation in the individuals older than 75 years, the age at which the incidence of dementia increases significantly [35]. We selected for probes that showed age-dependent increase or decrease of DNA modification (FDR corrected linear regression q < 0.05) within the top 10 % of the most variable probes, as measured by the coefficient of median absolute deviation, in the cerebral cortex and cerebellum (345 and 291 of 10,630 normalized CpG probes, respectively). The selected probes represented independent genes (rather than being clustered around a few selected genes), with an exception of a single gene (*BRAP*), which was represented by two probes in the cerebellum.

**Fig. 1:**
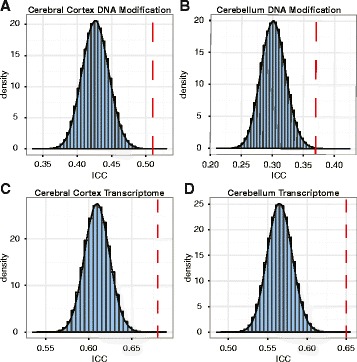
ICC analysis of DNA modification and gene transcription within the cerebral cortex and the cerebellum. The *histograms* represent the densities of the permuted mean ICC coefficients from samples of all ages and the *red dashed lines* show the mean ICC in the older individuals (>75 years). **a** Mean ICC of DNA modification in the cerebral cortex of older individuals (permuted *p* = 7 × 10^−6^). **b** Mean ICC of DNA modification in the cerebellum of older individuals (permuted *p* = 9 × 10^−4^). **c** Mean ICC of the transcriptome in the cerebral cortex of older individuals (permuted *p* = 5 × 10^−6^). **d** Mean ICC of the transcriptome in the cerebellum of older individuals (permuted *p* < 10^−6^)

The cortex showed significantly higher ICCs in the older individuals (>75 years; N = 85) compared with the permuted null distribution of ICCs that was generated by randomly subsampling 85 individuals from all samples (N = 348) across all ages (mean ICC ± standard deviation (SD) 0.51 ± 0.24 and 0.42 ± 0.02, respectively; permuted *p* = 7 × 10^−6^ (note that permutation usually involves relabeling of samples; however, random sampling of individuals equal to the sample size of the older individuals is, in effect, the same as relabeling the age of the samples and, therefore, we will refer to this method as permutation henceforth) (Fig. 1a). Similarly, significant differences were also detected in the cerebella of the older individuals (>75 years; N = 82) compared with the permuted data derived from 324 individuals (mean ICC ± SD 0.37 ± 0.29 and 0.30 ± 0.02, respectively; permuted *p* = 9 × 10^−4^; Fig. 1b). Consistent with these findings, a large proportion of the probes selected for the analysis showed significantly lower variance in the older individuals compared with the younger (<75 years) individuals in both the cortex and the cerebellum (60.0 % and 52.9 % of the selected probes, respectively, in comparison with 5 % expected by chance; Additional file 2: Figure S1a, b).

Next, we used the brain transcriptomic dataset to determine if the age dynamics are similar to the one observed in the DNA modification analysis. We selected the top 10 % of the most variable mRNAs within the cerebral cortex and the cerebellum (4880 of 48,803 normalized mRNA transcripts). Consistent with the epigenomic findings, we observed higher ICCs within the cortex tissues of older individuals (>75 years; N = 94) in comparison with the permuted data derived from 445 individuals (mean ICC ± SD 0.68 ± 0.14 and 0.61 ± 0.01, respectively; permuted *p* = 5 × 10^−6^; Fig. 1c). Likewise, the cerebellum samples showed higher ICCs within the older group (>75 years; N = 97) in comparison with the permuted data derived from 454 individuals (mean ICC ± SD 0.65 ± 0.16 and 0.56 ± 0.02, respectively; permuted *p* < 10^−6^; Fig. 1d). Again, the probes selected for the ICC analysis showed significantly lower variance in the older samples compared with the younger cohort (<75 years) in both the cortex and the cerebellum (40.5 % and 47.6 % of the selected probes, respectively, in comparison with 5 % expected by chance; Additional file 2: Figure S1c, d).

**Fig. 2:**
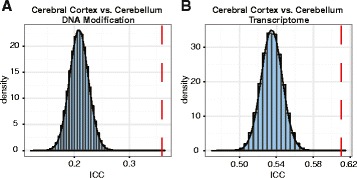
Loss of tissue-specific DNA modification and gene transcription patterns in the aging brain. The *histograms* represent the densities of the permuted mean ICC coefficients between two different brain regions (cerebral cortex and cerebellum) from samples of all ages: **a** DNA modification (permuted *p* < 10^−6^); **b** transcriptome (permuted *p* < 10^−6^). The *red dashed lines* show the mean cortex–cerebellum ICCs in the older individuals (>75 years)

Finally, we examined cortex–cerebellum DNA modification differences for possible diminished brain regional specificity. To explore this, we selected 112 probes that were commonly represented in both the cortex and the cerebellum analyses and performed multiple pair-wise ICC between the cerebral cortex and the cerebellum. The older brains (>75 years; N = 85 cortex and N = 82 cerebellum samples) showed significantly higher cortex–cerebellum similarity compared with the permuted data from the cortex (subsampling N = 85 of 348) and the cerebellum (subsampling N = 82 of 324) tissues (mean ICC ± SD 0.36 ± 0.32 and 0.21 ± 0.02, respectively; permuted *p* < 10^−6^; Fig. 2a). Similar analysis performed on 2661 mRNA transcripts, using the same criteria as above, showed significantly higher cortex–cerebellum similarity in the older brains (>75 years; N = 94 from cortex and N = 97 from cerebellum) compared with the permuted data derived from all samples consisting of both tissue types (mean ICC ± SD 0.61 ± 0.16 and 0.53 ± 0.01, respectively; permuted *p* < 10^−6^; Fig. 2b).

### The dynamics of DNA modification in Alzheimer’s disease

Following the evidence that aging is associated with epigenetic brain assimilation and regional dedifferentiation, we explored these phenomena in Alzheimer’s disease (AD), a disease for which old age is the primary risk factor [36]. Briefly, we performed epigenome-wide DNA modification profiling of brain samples collected from two monozygotic (MZ) twin sets and two dizygotic (DZ) twin sets (N = 8 individuals in total) who were participants in the Duke Twins Study of Memory in Aging and the National Academy of Sciences-National Research Council (NAS-NRC) Registry of World War II veteran male twins [37]. All co-twins exhibited differential age of AD onset. The earlier age of onset (EAO) twins were diagnosed with AD at 64.2 ± 5.7 years (mean ± SD) while the later age of onset (LAO) co-twins were diagnosed at 70.5 ± 6.5 years (mean difference in age of onset ± SD = 6.3 ± 8.6 years; Additional file 1: Table S1). We investigated three brain samples from each twin set: frontal cortex samples from both twins and one cerebellum sample from one of the twins. The cerebellum samples were matched for disease onset (i.e., two were LAO and two were EAO). DNA modification profiles were interrogated using the Human CpG island 12.1 K microarrays [38].

Locus-by-locus analysis identified 82 differentially modified loci in the cortex of EAO twins compared with their LAO co-twins (weighted *t*-test, nominal *p* < 0.05; Additional file 3: Table S2). In comparison, cerebral cortex versus cerebellum revealed 702 significant loci (weighted *t*-test, nominal *p* < 0.05), which is consistent with previous findings of major epigenetic differences between the cerebral cortex and the cerebellum [39].

For the AD cortex–cerebellum dedifferentiation analysis, we selected the top 5 % of the most differentially modified loci between the EAO and the LAO cortex (226 of 4523 unique loci represented on the 12.1 K microarray). EAO AD patients’ cortex (N = 4) modification profiles were more similar to the cerebellum in comparison with the null distribution permuted by random subsampling from eight LAO and EAO cortex samples (mean pair-wise ICC ± SD 0.48 ± 0.08 and 0.42 ± 0.03, respectively; permuted *p* = 0.014; Additional file 4: Figure S2a). Conversely, the bottom 5 % (i.e., the least differentially modified loci), which we used as a negative control, revealed no cortex–cerebellum difference between the EAO and the permuted data (ICC ± SD 0.40 ± 0.18 and 0.40 ± 0.02, respectively; permuted *p* = 0.51; Additional file 4: Figure S2b).

**Fig. 3:**
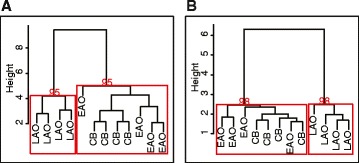
Unsupervised hierarchical clustering of DNA modification in the brains of EAO and LAO AD twins. **a** The *red boxes* indicate clades with higher than 80 % bootstrapping probability. Clustering, using the top 5 % of the most differentially modified loci, showed that cerebellum (*CB*) and EAO cerebral cortex form a single clade 95 % of the time while the cortex from the LAO co-twins are in a separate clade 95 % of the time. **b** In the top 82 AD onset-associated loci, the cerebral cortex of EAO twins and the cerebellum clustered into a single clade 98 % of the time, while LAO co-twins separated into a different clade 98 % of the time

We validated these findings using unsupervised hierarchical clustering and bootstrapping, which resulted in the LAO twins clustering together into one clade 95 % of the time, while cerebellum and EAO twins were grouped into a separate clade (Fig. 3a). These findings argue that the earlier onset and longer duration of AD may have accelerated the age-dependent epigenetic dedifferentiation of the brain into a more primal cerebellum-like state. Due to the small number of AD samples, we could not perform ICC analysis separately on cerebral cortex or cerebellum.

The same analysis was applied to the aforementioned 82 most differentially modified AD-onset associated loci (nominal *p* < 0.05). EAO AD patients’ modification patterns were more similar to the cerebellum in comparison with the AD cortices that were selected at random (mean ICC ± SD 0.50 ± 0.06 and 0.41 ± 0.04, respectively; permuted *p* < 10^−6^; Additional file 4: Figure S2c; Additional file 5: Figure S3a). Again, unsupervised hierarchical clustering and bootstrapping resulted in the EAO twins and the cerebellum clustering together into one clade 98 % of the time (Fig. 3b).

The changes observed in the AD brain samples, and perhaps in the aging brains in general [40], could be related to disproportional loss of neurons compared with neuroglia observed in AD [41]. To partially address this issue, we investigated buccal epithelium samples of AD twins. Both brain and buccal epithelium cells derive from a common germinal epithelium (ectoderm), yet buccal epithelial cells are far more homogenous in terms of their cellular composition. We performed ICC-based analyses of the buccal samples collected from 13 MZ and DZ twin pairs from the Duke Twins Study of Memory in Aging, who were discordant for AD at the time of sample collection (mean duration of illness for the affected twin at the time of collection ± SD = 1.7 ± 1.6 years; for more details see Additional file 1: Table S1). The top 5 % most differentially modified loci between the AD twins and their co-twins showed increased similarity amongst the AD twins (N = 13) from different pairs in comparison with the permuted data, which were derived by randomly sampling from 26 affected and unaffected twins (mean ICC ± SD 0.89 ± 0.05 versus 0.80 ± 0.03, respectively; permuted *p* = 1.4 × 10^−3^; Additional file 4: Figure S2d). The bottom 5 % (the least differentially modified loci as a negative control) showed no difference between the AD twins and their co-twins (mean ICC ± SD 0.83 ± 0.08 versus 0.83 ± 0.02, respectively; permuted *p* = 0.43; Additional file 4: Figure S2e). Using the 82 AD onset-associated loci from the brain study, we found that AD patients from unrelated twin pairs were approaching statistical significance in terms of similarity to each other in comparison with a randomly selected subset of samples (mean ICC ± SD 0.87 ± 0.06 versus 0.83 ± 0.02, respectively; permuted *p* = 0.07; Additional file 4: Figure S2f; Additional file 5: Figure S3b). These findings provide further evidence for age-associated epigenetic assimilation and suggest that this may not be limited to the brain but may also be present in other tissues of ectodermic origin or perhaps organism-wide.

In summary, our findings suggest that aging may be accompanied by loss of epigenetic uniqueness and tissue dedifferentiation, which may be exacerbated in AD. This implies that epigenetic drift, which leads to divergence of epigenetic patterns across individuals, is not an indefinite process.

### Gene Ontology enrichment in the aging brain and AD

To identify systematic aberrations of specific biological processes, we performed Gene Ontology (GO) enrichment analyses on all of the aging loci and the AD onset-associated loci identified in the earlier analyses. In the aging methylome, we found enrichment of terms such as prostaglandin and prostanoid metabolic process, as well as other lipid metabolic and biosynthesis processes, that could be related to aging and AD (Additional file 6: Table S3). Also enriched were neurodevelopment-related terms, including regulation of neurogenesis and cell projection organization. The transcriptome data showed enrichment of terms related to immunity, such as positive regulation of immune system process and immune response-regulating signaling pathway, which have also been associated with the aging brain and AD, as well as ontogenic terms like regulation of cell migration and regulation of developmental process. Lastly, in the AD onset-associated loci, we found enrichment of AD-relevant terms, such as regulation of caspase activity and lipid metabolic process, amongst a number of development related terms.

### Evidence that DNA modification and transcriptomic domains expand in the aging brain

Following the evidence that showed distant CpG methylation may become more concordant with age [32], as well as a loss of topologically associated domains while gaining cross-boundary interactions in senescent cell cultures [33], we investigated if domain boundaries change in older human brains and buccal cells.

To examine the change in domain boundaries, we estimated correlations between the loci of interest and its nearest neighbors as organized by their chromosomal coordinates in all previously tested datasets. A co-regulated domain was defined as three or more transcripts or DNA modification loci with consecutive Pearson’s correlation coefficient of 0.3 (generally accepted cutoff for weak positive correlation) or greater between nearest neighboring probes.

**Fig. 4:**
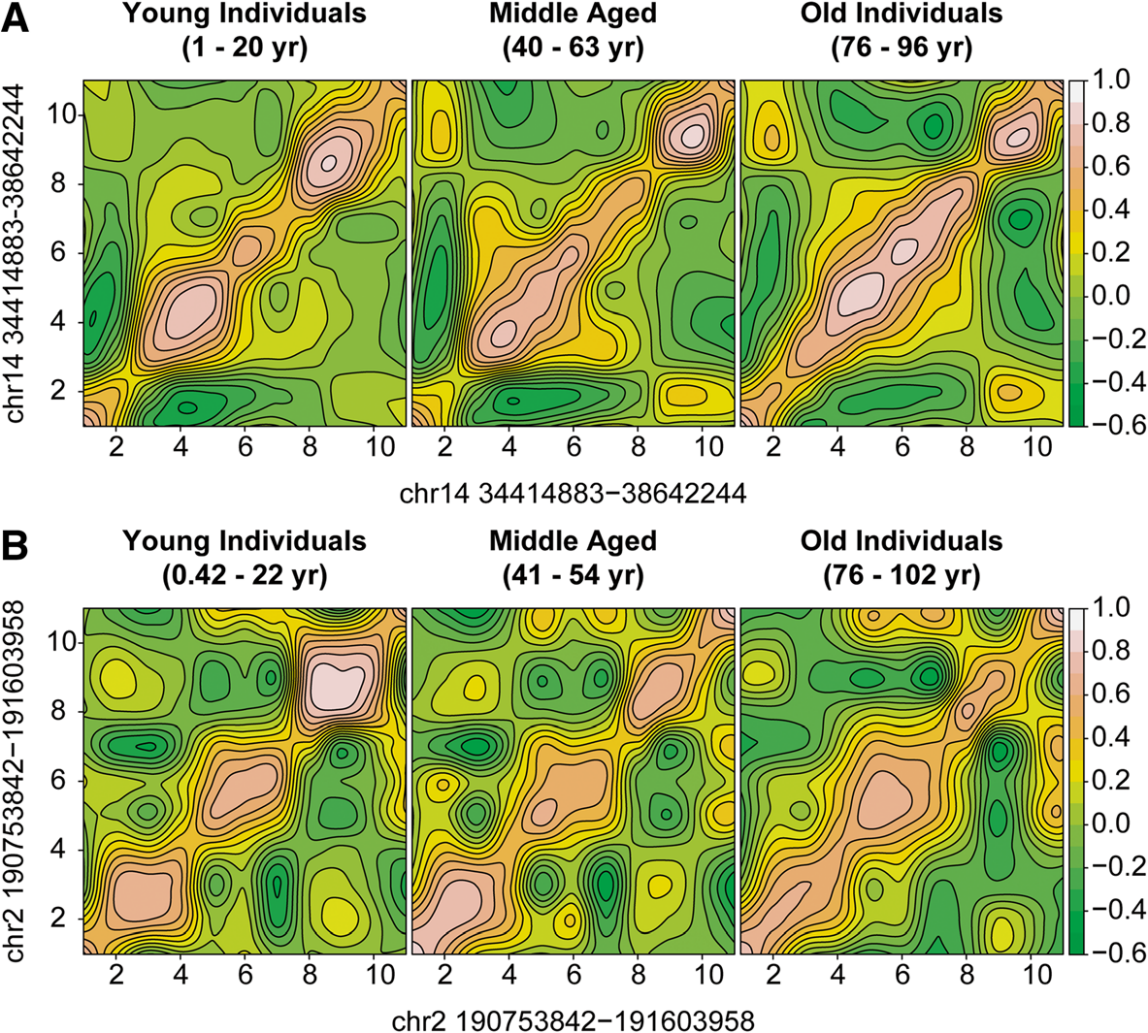
Examples of expanding DNA modification and transcriptomic domains. The contour plot represents the correlation coefficients between the nearest neighboring probes, where high correlation (r = 1) is represented in *white*, no correlation (r = 0) in *light green*, and anti-correlation (r = −1) in *dark green*. **a** The plot shows correlations between 11 DNA modification probes representing a ~4 Mb region on chr14: 34,414,883–38,642,244. Three distinct domains with high inter-probe correlation were detected in the young individuals (1–20 years), while the boundaries of these domains merged in the old individuals (76–96 years). The middle-aged individuals (40–63 years) showed an intermediate pattern. **b** The plot shows correlations between 11 transcripts representing a ~1 Mb region on chr2:190,753,842–191,603,958. Like the DNA modification data, the young individuals show distinct transcriptomic boundaries that diminished with age

We found larger DNA modification domain sizes in the cortex samples from older individuals (>75 years; N = 85) compared with the permuted data derived from 348 individuals (all comparisons represent the mean number of interacting loci per domain ± SD: 3.29 ± 0.64 and 3.20 ± 0.04, respectively; permuted *p* = 0.01; Fig. 4a; Additional file 7: Figure S4a; Additional file 8: Table S4). Similarly, the transcriptional domain sizes were larger in the older cortex samples (>75 years; N = 94) compared with the permuted data derived from 445 individuals (3.41 ± 0.79 and 3.35 ± 0.03, respectively; permuted *p* = 0.01; Fig. 4b; Additional file 7: Figure S4c; Additional file 8: Table S4). Further evidence for loss of domain structure was also detected in the AD twin brain samples. The EAO AD cortex samples (N = 4) demonstrated larger domains than the permuted data (3.87 ± 1.39 and 3.70 ± 0.06 genes, respectively; permuted *p* = 0.015; Additional file 7: Figure S4e), while the LAO AD cortex (N = 4) samples showed no difference (3.70 ± 1.10 versus 3.70 ± 0.06 genes; permuted *p* = 0.51).

We did not find a significant deviation in DNA modification domain size in the cerebellum between the older (>75 years; N = 82) compared with the permuted data derived from 324 individuals (3.27 ± 0.61 and 3.22 ± 0.05, respectively; permuted *p* = 0.13; Additional file 7: Figure S4b). Likewise, the transcriptomic domains did not show a significant difference between the older cerebellum (>75 years; N = 97) compared with the permuted data derived from 454 individuals (3.37 ± 0.79 and 3.37 ± 0.03, respectively; permuted *p* = 0.55; Additional file 7: Figure S4d). Inconsistent with the prediction, buccal samples of AD twins (N = 13) had smaller domains compared with the permuted data of 26 samples (3.54 ± 0.91 and 3.72 ± 0.10, respectively; permuted *p* = 0.04; Additional file 7: Figure S4f).

## Discussion

The current study provides evidence for a new molecular phenomenon in aging cells and tissues. Despite the limited sample size and the sparse coverage of the DNA modification microarray platforms used in this study, we show that the uniqueness and specificity of DNA modification and gene expression, on both the tissue and the individual level, diminish with old age. Reduced inter-tissue variation in older brains points to a potential loss of molecular fidelity, or dedifferentiation, of brain regions and this effect appears more pronounced in AD patients. Inter-individual epigenetic assimilation was also detected in buccal samples from individuals affected with AD compared with their unaffected co-twins. We also observed loss of boundaries and merging of neighboring DNA modification and transcriptomic domains in the cerebral cortex over time. It is likely that inter-individual epigenetic assimilation and intra-individual tissue dedifferentiation are two facets of the same phenomenon, mediated by aging-related deterioration of chromatin structure that compromises nuclear organization and cellular identity.

The transition from epigenetic divergence to convergence may be partially explained by the bimodality of DNA modification in the human genome. Most CpGs are heavily modified [42] and an imperfect replication of DNA modification patterns during mitosis would lead to gradual loss of modified cytosines over time. CpG islands, on the other hand, are generally unmodified [43] and epigenetic drift within the islands is likely to accumulate modified cytosines, both drifting towards the middle. Such DNA modification changes are unlikely to occur in isolation and may also involve chromatin changes. Consistent with Chandra et al. [33], we detected increased domain size in the brains of older individuals. The buccal samples, however, did not show an increase in domain size. Although the cause of the discrepancy is unclear, this could be related to the short duration of illness by AD twins at the time of sample collection or tissue-specific characteristics (i.e., rapidly dividing buccal cells versus mitotically arrested neurons and tissue-specific differences in DNA modification drift with age [44]).

Given that epigenetic assimilation was consistently detected in the cerebral cortex, which contains a substantial proportion of neurons [45], loss of DNA modification fidelity during replication is unlikely to be the sole mechanism behind epigenetic drift. Studies have shown that 5-hydroxymethylcytosine accounts for up to 40 % of modified cytosines in the brain [46, 47]. Active demethylation via ten-eleven translocation (TET) enzymes and thymine DNA glycosylase (TDG) were shown to play a critical role in epigenetic reprogramming of cells [48–50]. Therefore, aberrations in the active DNA demethylation and remethylation pathways may contribute to epigenetic drift and other age-dependent epigenetic changes in the brain.

Our DNA modification and transcriptomic studies suggest that AD may be related to accelerated aging of the brain. In fact, several studies have already shown that plaques and tangles, marked features of AD, also form in the normal aging brain but to a lesser extent [51]. It has also been suggested that neurodegenerative diseases may be caused by the loss of cellular maintenance over time [52, 53], which is consistent with the compromised chromatin domain integrity observed in this study. In addition to enrichment of disease-associated GO terms in the AD samples, the aging brain also showed enrichment of terms closely linked to AD, such as immune response and lipid metabolic pathways (e.g., prostaglandins; reviewed in [54]), which further corroborates the connection between the two. We also observed enrichment of terms related to developmental processes in all samples, suggesting a possible link between tissue dedifferentiation and ontogeny. However, the weight of our AD findings, especially in the brain study, is severely limited by its small sample size and our preliminary findings should be further explored in larger datasets.

The precise molecular origin and biological implications of age-dependent epigenetic assimilation and dedifferentiation warrant further exploration. While the increase in domain size, mediated by the compromised integrity of domain boundaries, provides an elegant explanation of the phenomenon, other alternatives have to be considered. For instance, ascertainment bias may simulate epigenetic assimilation. As excessive epigenetic drift may predispose an individual to a fatal disease, such as cancer, inter-individual similarity amongst the older individuals could be due to the survivorship of less-deviant individuals. Another explanation for molecular assimilation in the brain can be age-dependent changes in the proportion of glial cells to neurons. For instance, senescent microglia can manifest an overactive immune response that results in neurotoxicity in the aging brain, causing changes in neuron to glia ratios, where glia could become the dominant cell type sampled in older brains (reviewed in [40]). Furthermore, if epigenetically diverse cells die faster than epigenetically more similar cells, this could result in evidence for false similarity.

Currently, the best solution available to address the cellular heterogeneity problem is either by flow sorting the cells to neurons/glia prior to the experiment or to use a computation algorithm built using sorted cells to estimate the contribution from the two cellular fractions [55]. However, DNA modification and transcriptomic patterns vary significantly even within neurons [56] and likely the same applies to glial cells. Therefore, separation of only neurons and glia, either experimentally or computationally, may be insufficient to effectively differentiate brain cellular effects from the genuine epigenetic ones. In addition, the prediction made by the computational algorithm also depends on the quality of the data (i.e., quality of tissue biopsy, microarray batch effect, etc.), which adds another layer of complexity. Nevertheless, evidence for assimilation in buccal cells suggests that our findings are not purely due to changes in cell composition; though the confounding effects of cellular composition should be explored in greater detail.

It has been proposed that stochastic epigenetic drift may increase fitness in changing environments [24]. On the other hand, epigenetic deviations may also cause or predispose to a disease. Therefore, epigenetic drift in aging may be closely tied to the concept of antagonistic pleiotropy [57]; that is, a trait which increases fitness but can become detrimental later in life [58]. In this connection, assimilation and dedifferentiation could be simply viewed as an extension of this process. This could imply that our observations are the result of a predestined state of cells in the later stages of life that initially served a physiological or an evolutionary function.

## Conclusions

Our study shows that drift-mediated increase in inter-individual variability may be finite. This phenomenon is concurrent with an intra-individual, age-dependent increase in similarity between the cortex and the cerebellum. Similarly, though limited in sample size, investigations using twin samples discordant for AD corroborated instances of epigenetic assimilation and dedifferentiation. However, these observations must be approached with vigilance given the potential for ascertainment bias and confounding cellular composition changes that can occur with old age. Since such confounders are difficult to control in humans, dedicated animal experiments investigating the late stages of life are warranted to confirm our findings. Lastly, it is pertinent to uncover the differences and similarities between developmental differentiation and age-dependent dedifferentiation. This question is particularly important for the concept of epigenetic rejuvenation [59]. It postulates that if age-related epigenetic changes could be disentangled from developmental programming, reversal of cellular senescence without altering cellular identity may be possible. Under this model, it is assumed that epigenetic factors determining the cell and tissue specificity remain unchanged during aging. Our findings, however, suggest that aging does affect cell-specific epigenomes; therefore, cellular rejuvenation may require re-establishing cellular identity.

## Methods

### Samples

We obtained post-mortem brain frontal cortex tissues from two pairs of MZ twins and two pairs of DZ twins with a diagnosis of AD, as well as cerebellum tissues from one of each pair. The twins with earlier age of onset were classified as earlier-onset (EAO) AD while their co-twin, with later age of onset, was classified with later-onset (LAO) AD. The mean age at onset and age at collection for EAO twins were 64.2 ± 5.7 years (mean ± SD) and 77.0 ± 8.4 years, respectively, and 70.5 ± 6.5 years and 75.2 ± 8.6 years, respectively, for the LAO co-twins. We also obtained buccal samples from 13 MZ or DZ twin pairs who are discordant for AD at the time of sample collection (mean age at onset = 77.8 ± 2.1 years; mean age at collection = 79.2 ± 1.9 years). All twins were participants in the Duke Twins Study of Memory in Aging.

The North American Brain Expression Consortium and UK Human Brain Expression Database (Gene Expression Omnibus (GEO) accession GSE36192) consisted of 911 cerebral cortex and cerebellum samples from age 0.42 to 102 with mean age (mean ± SD) of 48.8 ± 25.6 years. The North American Brain Expression Consortium: DNA Methylation (GEO accession GSE36194) had 724 cerebral cortex and cerebellum samples between ages 0.42 and 102, with mean age of 48.4 ± 27.7 years. More detailed information on the samples can be found in Additional file 1: Table S1.

### Microarray experiment

Genomic DNA from twin brain cortex, cerebellum, and buccal samples was extracted using standard proteinase K digestion followed by phenol chloroform purification. Microarray experiments were performed using a common reference design, where the unmethylated fraction of genomic DNA for each individual is end-labeled with Cy3 dye and subjected to hybridization at 42 °C for 16 h against a common reference pool DNA labeled with Cy5 dye [60]. The labeled DNA was hybridized onto the human CpG Island 12.1 K microarray [38], consisting of 12,192 clones representing CG-rich elements across the genome. All microarray experiments were performed in two technical replicates. Microarrays were scanned on an Axon 4000b scanner using Genepix 6.0 software.

### Public gene expression/methylation microarray

We utilized a large gene expression microarray dataset (N = 911) from the North American Brain Expression Consortium and UK Human Brain Expression Database (GEO accession GSE36192; http://www.ncbi.nlm.nih.gov/geo/query/acc.cgi?acc=GSE36192) and DNA modification dataset (N = 724) from the North American Brain Expression Consortium: DNA Methylation (GEO accession GSE36194; http://www.ncbi.nlm.nih.gov/geo/query/acc.cgi?acc=GSE36194). Cortex and cerebellum samples were normalized separately using robust quantile normalization and background corrected using Robust Multiarray Average (RMA) background correction, using the “preprocessCore” package for R [61]. Hierarchical clustering was performed using all normalized expression values and outliers (samples indicated as an outgroup in the dendrogram) were removed from all analyses. The methylation probes were further processed to check for probes containing single-nucleotide polymorphisms (SNPs) with a minor allele frequency of 5 % that lie within 5 bp of the target CpG site. We found that only 0.66 % of probes used in the analysis met these criteria (SNP data from the Infinium HD Methylation SNP List at the vendor’s website). Finally, the probes were trimmed to include the top 10 % of most variable loci within each tissue, as measured by coefficient of median absolute deviation, or the top 10 % of the most differentially modified regions between the brain cortex and the cerebellum.

### Alzheimer’s disease microarray

The microarrays were normalized using loess and R quantile normalization and background corrected using normexp background correction in the “limma” package in R [61–63]. Redundant microarray probes, as well as probes containing repetitive sequences or probes that cannot be mapped to a genomic coordinate, were removed from all analyses; the total number of probes analyzed was 4523 (of 12,192) after trimming. Locus-by-locus DNA modification changes between groups were compared using limma F-test or weighted *t*-test (for two groups) and subjected to correction for multiple testing by the Benjamini–Hochberg FDR.

For the trimming of the AD dataset for the most variable loci, we included the top 5 % of the most variable loci, as measured by coefficient of median absolute deviation, to account for reduced power due to smaller sample size in comparison with the public datasets.

### Intra-class correlation analyses and permutation

Intra-class correlation (ICC) was performed using the “IRR” package in R [61]. More specifically, we used a one-way model with row effects, where the n × m matrix has n probes and m subjects (i.e., m = 2 for pairwise ICC). The ICC coefficient (*R*) was estimated by: $R\kern0.5em =\kern0.5em \frac{M{S}_{bet}\kern0.5em -\kern0.5em M{S}_{within}}{M{S}_{bet}\kern0.5em +\kern0.5em \left(m-1\right)M{S}_{within}}$, where MS_bet_ is the mean square between subjects, MS_within_ is the mean square within subjects, and m is the number of subjects. We calculated the fraction of total variation of the n × m data points that is due to between rows. Unsupervised hierarchical clustering/approximately unbiased bootstrapping [64] was performed using the “pvclust” [65] package in R [61]. Lastly, a permutation test was performed by randomly sampling (100,000–1,000,000 times) N samples (where N = the same number of individuals as the number of individuals older than 75 years) from a pool of all individuals to calculate the mean ICC or domain size and generate the null distribution to which the actual mean ICC or domain size was compared against. Gene Ontology (GO) enrichment was performed using GO-Elite [66].

### DNA modification and transcriptomic domain size analysis

DNA modification and transcriptomic domain size analysis was performed by using pair-wise Pearson’s correlation between the target region and its nearest neighbors as defined by their chromosomal coordinates. More specifically, the correlation coefficient was calculated by comparing a list of values for a given probe (i.e., probe × with N data points, where N is the number of individuals in a group) to its nearest neighboring probe from the same group of individuals. A domain was defined as regions with a consecutive correlation coefficient of 0.3 (generally accepted cutoff for weak positive correlation) or greater, involving more than three loci within each chromosome. Sex chromosomes were excluded from the analysis.

### Availability of supporting data

The datasets supporting the results of this article (Alzheimer’s disease dataset) are available in the GEO repository (accession GSE61242; http://www.ncbi.nlm.nih.gov/geo/query/acc.cgi?acc=GSE61242).

### Ethics statement

All procedures were approved by the Centre for Addiction and Mental Health Research Ethics Board (175/2009) and the Duke Institutional Review Board (Pro00011940, Pro00009028, and Pro00011934). All experiments were performed in accordance with relevant guidelines, regulations, and the Declaration of Helsinki. Informed consent was obtained from all participants or their legal representatives.

## Additional files

Additional file 1: Table S1. Sample information. (PDF 40 kb) Additional file 2: Figure S1. Examples of data distribution for DNA modification and transcriptome data in older (>75 years) and younger (>75 years) individuals. Violin plots showing representative densities of DNA modification (beta values) and probe signal intensities in transcriptome data for older and younger individuals for a given probe. One-tailed F-test was used to identify cases where older individuals had lower variance than the young (i.e., F-test *p* < 0.05). **a** An example of a DNA modification probe where the older individuals had significantly lower variance than the younger individuals. **b** An example of a DNA modification probe where older individuals did not show significantly smaller variance compared with the younger individuals. **c** An example of a transcript with smaller variance in older compared with younger individuals. **d** An example of a transcript with non-significant difference of variance. (PDF 410 kb) Additional file 3: Table S2. List of significant loci (*p* < 0.05) in the cerebral cortex of twins different for AD age of onset. (PDF 73 kb) Additional file 4: Figure S2. Permuted null distribution of mean ICC in AD twin samples. ICC densities of the permuted null from all samples compared with the mean ICC in the indicated subset sample of interest (*red dashed line*). **a** EAO cortex versus cerebellum using the top 5 % of differentially modified loci (permuted *p* = 0.014). **b** EAO cortex versus cerebellum using the bottom 5 % of differentially modified loci (permuted *p* = 0.51). **c** EAO cortex versus cerebellum using the nominally significant (*p* < 0.05) DNA modification loci (permuted *p* < 10^-6^). **d** AD affected versus unaffected co-twin buccal samples using the top 5 % of differentially modified loci (permuted *p* = 1.4 × 10^-3^). **e** AD affected versus unaffected co-twin buccal samples using the bottom 5 % of differentially modified loci (permuted *p* = 0.43). **f** AD affected versus unaffected co-twin buccal samples using the nominally significant (*p* < 0.05) DNA modification loci from the cortex (permuted *p* = 0.07). (PDF 441 kb) Additional file 5: Figure S3. ICC of DNA modification for the 82 disease-specific differentially modified loci (*p* < 0.05). The Z-scores are normalized ICC coefficients, where positive Z-scores represent ICC coefficients that are higher than the mean, while negative Z-scores show the opposite. Cumulative Z-scores for normalized ICC coefficients represent the following: 0 % includes none, 50 % includes half, and 100 % includes all Z-scores. **a** Approximately 90 % of Z-scores are positive in the comparison of the EAO AD cortex samples versus cerebellum (*CB*) but only ~25 % of Z-scores are positive in the LAO versus cerebellum, indicating a more advanced state of dedifferentiation for EAO AD cortices. LAO versus EAO show an even distribution. **b** Buccal cell analysis showed that ~75 % AD twins, but only ~50 % of their healthy co-twins, had higher than average similarity as measured by Z-scores of ICC. (PDF 123 kb) Additional file 6: Table S3. Gene Ontology (GO) enrichment analysis of the age and AD onset-associated probes. (XLS 54 kb) Additional file 7: Figure S4. Permuted null distribution of mean domain length. The *histogram* represents the densities of the permuted null distribution from all samples and the *red dashed line* is the mean domain length in the indicated subset sample of interest (i.e., older individuals (>75 years), EAO cortex, or AD buccal cells). **a** Mean DNA modification domain length of older individual in the cerebral cortex (permuted *p* = 0.01). **b** Mean DNA modification domain length of older individual in the cerebellum (permuted *p* = 0.13). **c** Mean domain length of older individual transcriptome in the cerebral cortex (permuted *p* = 0.01). **d** Mean domain length of older individual transcriptome in the cerebellum (permuted *p* = 0.51). **e** Mean domain length of the EAO cerebral cortex (permuted *p* = 0.015). **f** Mean domain length of the AD-affected twin buccal samples (permuted *p* = 0.04). (PDF 131 kb) Additional file 8: Table S4. Raw correlation matrix of DNA modification and transcriptome data for young, middle aged, and old individuals used for Fig. 4. (PDF 111 kb)
