# Supplementary material for: Epigenetic assimilation in the aging human brain
Source: Genome Biol. 2016 Apr 28;17:76. doi: 10.1186/s13059-016-0946-8 (PMC4848814; doi:10.1186/s13059-016-0946-8)
Supplement: Additional file 1: Table S1. — Sample information. (PDF 40 kb) [file 13059_2016_946_MOESM1_ESM.pdf]

SI Table S1. Sample information.

Description of the AD twin samples				
Tissue Type	Status	Zygosity	Age at Onset (mean±SD)	Age at Collection (mean±SD)
Cortex	Earlier Onset AD	2MZ/2DZ	65, 72, 59, 61 (64.2±5.7)	82, 86, 68, 72 (77.0±8.4)
	Later Onset AD	2MZ/2DZ	75, 77, 66, 64 (70.5±6.5)	81, 84, 70, 66 (75.2±8.6)
Cerebellum	Earlier Onset AD	1MZ/1DZ	65, 72 (68.5±5.0)	82, 86 (84±2.8)
	Later Onset AD	1MZ/1DZ	66, 64 (65±1.4)	70, 66 (68±2.8)
Buccal	AD	6MZ/7DZ	77.8±2.1	79.2±1.9
	Unaffected	6MZ/7DZ	N/A	79.8±2.2

Description of the steady state brain mRNA samples					
Tissue Type	N	Age (years)	Age (mean±SD)	Sex	PMI (mean±SD)
Cortex	455	0.42-102	48.8±25.7	145F/310M	21.1±19.7
Cerebellum	456	0.42-102	48.8±25.6	144F/312M	21.2±19.7

Description of the steady state brain methylation samples					
Tissue Type	N	Age (years)	Age (mean±SD)	Sex	PMI* (mean±SD)
Cortex	369	0.42-102	48.7±25.8	43F/90M/236U**	14.1±5.3
Cerebellum	355	0.42-102	48.4±27.8	35F/86M/234U**	13.9±5.4

*based on available sample information

**U = Unknown; information unavailable
