# Supplementary material for: Epigenetic assimilation in the aging human brain
Source: Genome Biol. 2016 Apr 28;17:76. doi: 10.1186/s13059-016-0946-8 (PMC4848814; doi:10.1186/s13059-016-0946-8)
Supplement: Additional file 2: Figure S1. — Examples of data distribution for DNA modification and transcriptome data in older (>75 years) and younger (>75 years) individuals. Violin plots showing representative densities of DNA modification (beta values) and probe signal intensities in transcriptome data for older and younger individuals for a given probe. One-tailed F-test was used to identify cases where older individuals had lower variance than the young (i.e., F-test p < 0.05). a An example of a DNA modification probe where the older individuals had significantly lower variance than the younger individuals. b An example of a DNA modification probe where older individuals did not show significantly smaller variance compared with the younger individuals. c An example of a transcript with smaller variance in older compared with younger individuals. d An example of a transcript with non-significant difference of variance. (PDF 410 kb) [file 13059_2016_946_MOESM2_ESM.pdf]

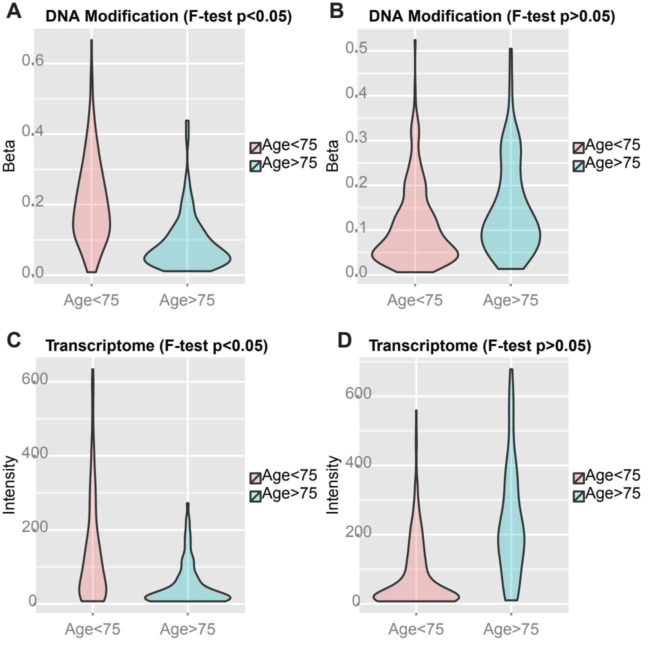
A
DNA Modification (F-test p<0.05)
B
DNA Modification (F-test p>0.05)
C
Transcriptome (F-test p<0.05)
D
Transcriptome (F-test p>0.05)
Beta
Intensity
0.0
0.2
0.4
0.6
0.1
0.3
0.5
0
200
400
600
Age<75
Age>75
