# Supplementary material for: Epigenetic assimilation in the aging human brain
Source: Genome Biol. 2016 Apr 28;17:76. doi: 10.1186/s13059-016-0946-8 (PMC4848814; doi:10.1186/s13059-016-0946-8)
Supplement: Additional file 3: Table S2. — List of significant loci (p < 0.05) in the cerebral cortex of twins different for AD age of onset. (PDF 73 kb) [file 13059_2016_946_MOESM3_ESM.pdf]

SI Table S2. List of significant loci ($p<0.05$) in the cerebral cortex of twins different for AD age of onset.

UHNID	Genome Location	Distance (bp)	Gene Symbol	GeneID
UHNhscpg0005706	chr8:8860048-8860910	0	THEX1	90459
UHNhscpg0004893	chr19:6862929-6863133	13637	SIX3	6496
UHNhscpg0005497	chr4:174254525-174255180	0	HMGB2	3148
UHNhscpg0011218	chr19:5953724-5953889	0	RANBP3	8498
UHNhscpg0009988	chr1:215521769-215522004	432361	MGMT	4255
UHNhscpg0008760	chr8:50538115-50538190	246752	FLJ45455	388336
UHNhscpg0005663	chr5:52081719-52081793	5002	FOXL2	668
UHNhscpg0008069	chr12:102092189-102092425	0	CHPT1	56994
UHNhscpg0008357	chr1:54355531-54355620	420456	SALL1	6299
UHNhscpg0011164	chr13:80251706-80251844	859047	IKZF2	22807
UHNhscpg0010960	chr3:2140036-2140938	0	CNTN4	152330
UHNhscpg0010408	chr5:161143932-161144010	154	OXR1	55074
UHNhscpg0010908	chr1:180736035-180736254	0	XPR1	9213
UHNhscpg0000208	chr1:11322016-11323073	0	FRAP1	2475
UHNhscpg0005543	chr11:44332091-44332260	15448	PAX2	5076
UHNhscpg0000577	chr14:37127633-37128348	0	PAX9	5083
UHNhscpg0006170	chr6:45219715-45219815	0	SUPT3H	8464
UHNhscpg0008762	chr7:8197140-8197186	0	ICA1	3382
UHNhscpg0000241	chr11:107461728-107462314	0	ELMOD1	55531
UHNhscpg0005982	chr6:144042375-144042464	0	PHACTR2	9749
UHNhscpg0001198	chr5:140729521-140730195	0	PCDHGA1	56114

UHNhscpg0010352	chr7:22862098-22862545	0	TOMM7	54543
UHNhscpg0000478	chr2:113299334-113299866	0	POLR1B	84172
UHNhscpg0011058	chr20:6103206-6103310	0	C20orf42	55612
UHNhscpg0009262	chr3:185326402-185326618	0	SENP2	59343
UHNhscpg0007672	chr14:51259096-51259859	0	NIN	51199
UHNhscpg0000646	chr15:72978212-72978938	0	HIGD2BP	123346
UHNhscpg0002102	chr6:25698651-25699132	0	SCGN	10590
UHNhscpg0008035	chr3:28768841-28769077	86140	DTNA	1837
UHNhscpg0000652	chr14:34145267-34145696	0	NPAS3	64067
UHNhscpg0011782	chr12:18869657-18869767	0	PLCZ1	89869
UHNhscpg0004272	chr7:157754896-157755094	0	PTPRN2	5799
UHNhscpg0009265	chr11:32605136-32605885	0	EIF3M	10480
UHNhscpg0005799	chr4:93612764-93612815	0	GRID2	2895
UHNhscpg0010180	chr3:32858778-32859102	248576	NR5A2	2494
UHNhscpg0002833	chr11:62445636-62446980	0	LOC51035	51035
UHNhscpg0006174	chr1:16175480-16175629	0	SPEN	23013
UHNhscpg0002145	chr1:116960860-116961486	0	C1orf203	84852
UHNhscpg0006151	chr14:54685966-54686339	107843	GRHL1	29841
UHNhscpg0007185	chr1:212874408-212874637	283355	MGC26647	219557
UHNhscpg0010094	chr10:114542866-114542933	0	VTI1A	143187
UHNhscpg0002850	chr17:75955635-75955946	33376	LRRC8B	23507
UHNhscpg0010101	chr10:22513913-22514088	733757	EPHA3	2042
UHNhscpg0008377	chr5:119750407-	347872	RALYL	138046

	119750559			
UHNhscpg0005205	chr20:44518785-44519558	0	NEURL2	140825
UHNhscpg0000830	chr20:11309478-11309784	19233	KIF11	3832
UHNhscpg0005280	chr8:114739195-114739321	4004	PTPN13	5783
UHNhscpg0011692	chr8:39629353-39629436	0	ADAM2	2515
UHNhscpg0004273	chr5:19655452-19655554	0	CDH18	1016
UHNhscpg0002088	chr2:71356722-71358044	0	MCEE	84693
UHNhscpg0007665	chr8:41511572-41511774	0	NKX6-3	157848
UHNhscpg0007259	chr1:211750802-211750993	0	SLC30A1	7779
UHNhscpg0004966	chr2:45164651-45165365	102909	TMEFF2	23671
UHNhscpg0009656	chr9:129400879-129401043	0	LMX1B	4010
UHNhscpg0005789	chr6:155500048-155500099	0	TIAM2	26230
UHNhscpg0005980	chr3:76941173-76941283	86892	CDS1	1040
UHNhscpg0008220	chr4:26858769-26859739	395938	CLINT1	9685
UHNhscpg0009919	chr3:143815424-143815518	15656	SOX2	6657
UHNhscpg0011078	chr16:33956373-33956680	70187	ALCAM	214
UHNhscpg0004168	chr20:7662145-7662336	134358	FOXD1	2297
UHNhscpg0001823	chr9:115983681-115983911	0	FKBP15	23307
UHNhscpg0004853	chr2:223288492-223288996	154101	MDGA2	161357
UHNhscpg0001018	chr20:11309478-11309784	77502	GNG11	2791
UHNhscpg0010587	chr20:32077436-32077771	328204	NAALADL2	254827
UHNhscpg0011447	chr17:28708184-28708307	0	CPD	1362
UHNhscpg0010087	chr1:48190779-48190856	292005	SOX6	55553

UHNhscpg0009864	chr4:4866664-4866900	350595	ADAMTSL1	92949
UHNhscpg0005920	chr1:6663435-6663620	105247	TMEM100	55273
UHNhscpg0009645	chr2:24148903-24149577	0	ATAD2B	54454
UHNhscpg0006093	chr13:85059344-85059622	203891	CFTR	1080
UHNhscpg0007153	chr16:47409483-47409531	0	ITFG1	81533
UHNhscpg0007521	chr18:47721421-47721819	0	MYO5B	4645
UHNhscpg0006473	chr9:4666184-4666739	0	C9orf68	55064
UHNhscpg0003372	chr13:97683225-97683322	58330	CXorf55	139804
UHNhscpg0010611	chr3:89377682-89377720	0	EPHA3	2042
UHNhscpg0005865	chr2:111878955-111879262	0	BCL2L11	10018
UHNhscpg0003952	chr7:5462215-5462374	0	KIAA1856	84629
UHNhscpg0007838	chr11:130488782-130489011	92077	HPYR1	93668
UHNhscpg0010801	chr4:109975432-109975499	0	COL25A1	84570
UHNhscpg0006064	chr5:110406357-110406632	0	TSLP	85480
UHNhscpg0010564	chr17:62223312-62223461	0	SNORD104	692227
UHNhscpg0010780	chr11:13434984-13435091	0	BTBD10	84280
