# Supplementary figures and images for: Epigenetic assimilation in the aging human brain
Source: Genome Biol. 2016 Apr 28;17:76. doi: 10.1186/s13059-016-0946-8 (PMC4848814; doi:10.1186/s13059-016-0946-8)

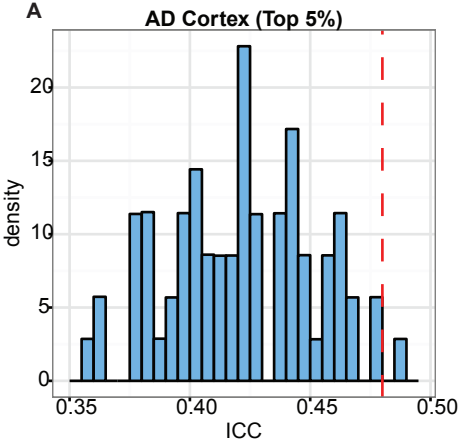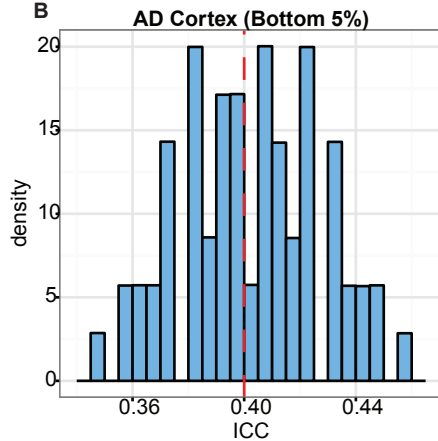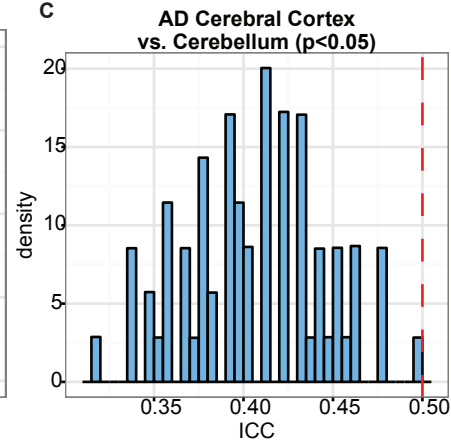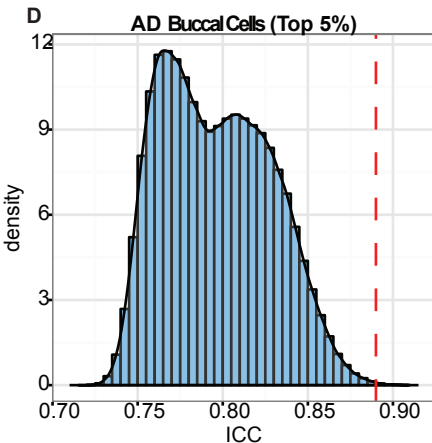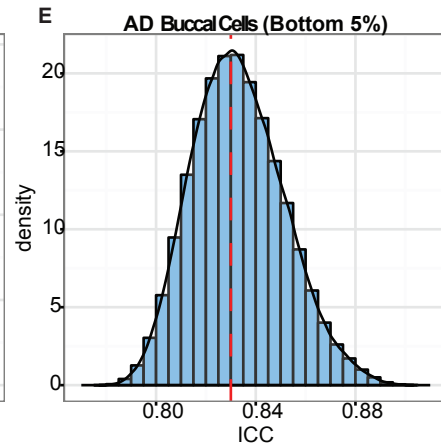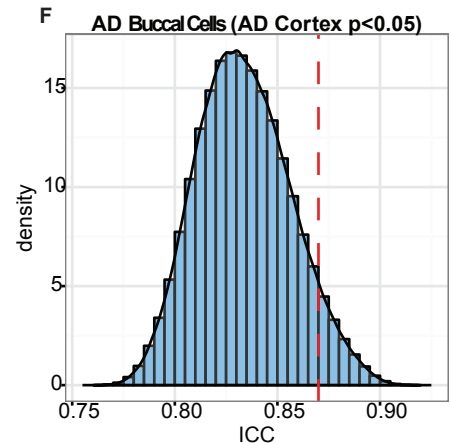

Supplement: Additional file 4: Figure S2. — Permuted null distribution of mean ICC in AD twin samples. ICC densities of the permuted null from all samples compared with the mean ICC in the indicated subset sample of interest (red dashed line). a EAO cortex versus cerebellum using the top 5 % of differentially modified loci (permuted p = 0.014). b EAO cortex versus cerebellum using the bottom 5 % of differentially modified loci (permuted p = 0.51). c EAO cortex versus cerebellum using the nominally significant (p < 0.05) DNA modification loci (permuted p < 10-6). d AD affected versus unaffected co-twin buccal samples using the top 5 % of differentially modified loci (permuted p = 1.4 × 10^-3^). e AD affected versus unaffected co-twin buccal samples using the bottom 5 % of differentially modified loci (permuted p = 0.43). f AD affected versus unaffected co-twin buccal samples using the nominally significant (p < 0.05) DNA modification loci from the cortex (permuted p = 0.07). (PDF 441 kb) [file 13059_2016_946_MOESM4_ESM.pdf]

**A**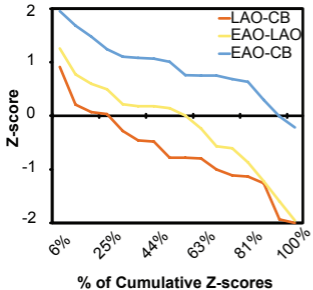**B**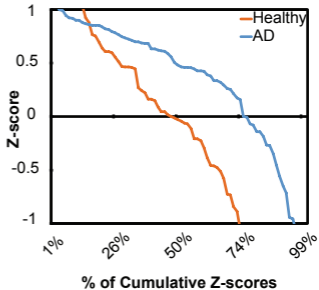

Supplement: Additional file 5: Figure S3. — ICC of DNA modification for the 82 disease-specific differentially modified loci (p < 0.05). The Z-scores are normalized ICC coefficients, where positive Z-scores represent ICC coefficients that are higher than the mean, while negative Z-scores show the opposite. Cumulative Z-scores for normalized ICC coefficients represent the following: 0 % includes none, 50 % includes half, and 100 % includes all Z-scores. a Approximately 90 % of Z-scores are positive in the comparison of the EAO AD cortex samples versus cerebellum (CB) but only ~25 % of Z-scores are positive in the LAO versus cerebellum, indicating a more advanced state of dedifferentiation for EAO AD cortices. LAO versus EAO show an even distribution. b Buccal cell analysis showed that ~75 % AD twins, but only ~50 % of their healthy co-twins, had higher than average similarity as measured by Z-scores of ICC. (PDF 123 kb) [file 13059_2016_946_MOESM5_ESM.pdf]

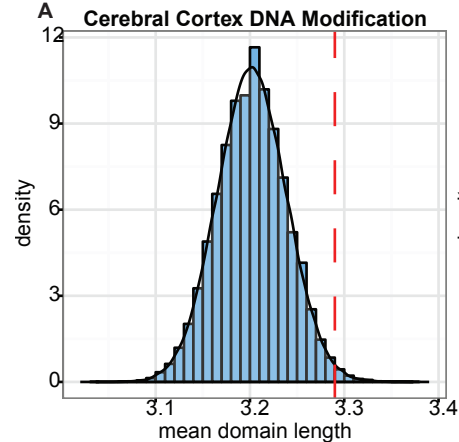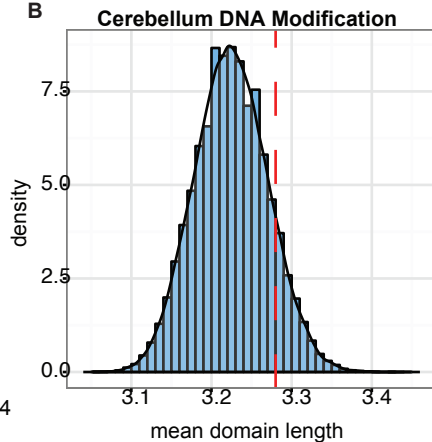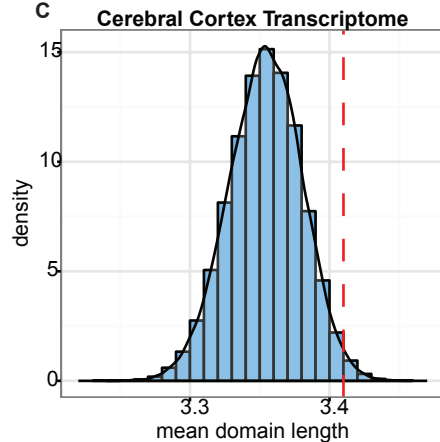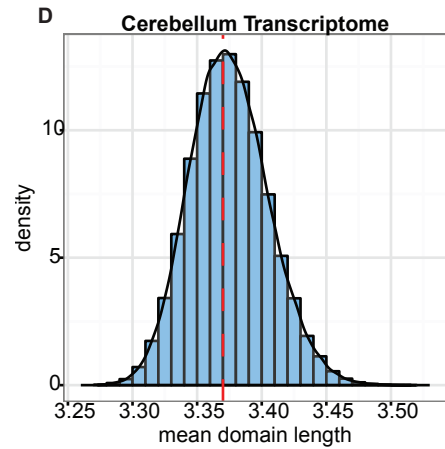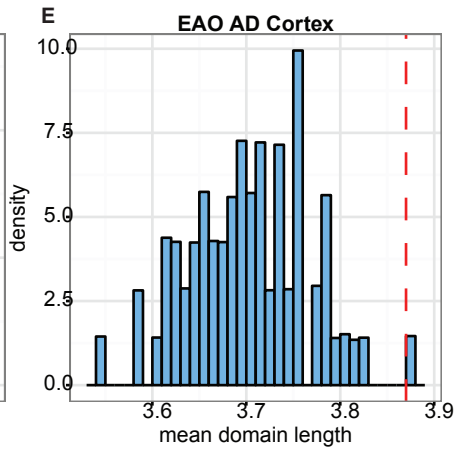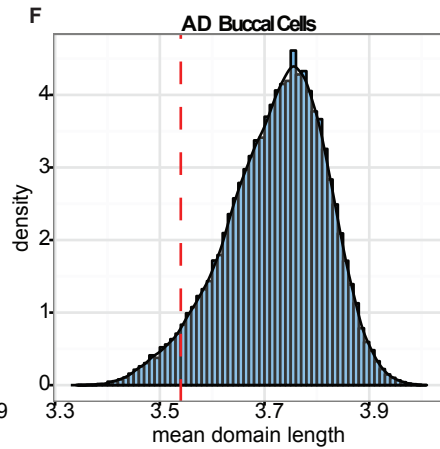

Supplement: Additional file 7: Figure S4. — Permuted null distribution of mean domain length. The histogram represents the densities of the permuted null distribution from all samples and the red dashed line is the mean domain length in the indicated subset sample of interest (i.e., older individuals (>75 years), EAO cortex, or AD buccal cells). a Mean DNA modification domain length of older individual in the cerebral cortex (permuted p = 0.01). b Mean DNA modification domain length of older individual in the cerebellum (permuted p = 0.13). c Mean domain length of older individual transcriptome in the cerebral cortex (permuted p = 0.01). d Mean domain length of older individual transcriptome in the cerebellum (permuted p = 0.51). e Mean domain length of the EAO cerebral cortex (permuted p = 0.015). f Mean domain length of the AD-affected twin buccal samples (permuted p = 0.04). (PDF 131 kb) [file 13059_2016_946_MOESM7_ESM.pdf]
