# Supplementary material for: Epigenetic assimilation in the aging human brain
Source: Genome Biol. 2016 Apr 28;17:76. doi: 10.1186/s13059-016-0946-8 (PMC4848814; doi:10.1186/s13059-016-0946-8)
Supplement: Additional file 8: Table S4. — Raw correlation matrix of DNA modification and transcriptome data for young, middle aged, and old individuals used for Fig. 4. (PDF 111 kb) [file 13059_2016_946_MOESM8_ESM.pdf]

SI Table S4. Raw correlation matrix of DNA modification and transcriptome data for young, middle aged, and old individuals used for Figure 4.

Correlation matrix for the methylome domain plot (Figure 4A)

	0.117	0.175	-0.228	-0.254	-0.231	-0.176	0.028	0.083	0.204	0.065	1.000	young
	-0.092	0.432	-0.364	-0.426	-0.236	-0.040	-0.065	-0.183	0.020	0.072	1.000	middle
**11**	0.023	0.233	-0.169	-0.254	-0.090	0.173	0.149	-0.196	0.085	-0.030	1.000	old
	-0.090	0.086	0.008	0.017	0.042	-0.001	-0.271	0.138	0.522	1.000	0.065	
	0.169	0.250	-0.167	-0.324	-0.264	-0.116	-0.069	-0.053	0.653	1.000	0.072	
**10**	0.119	0.292	-0.072	-0.476	-0.248	-0.360	-0.439	-0.348	0.564	1.000	-0.030	
	-0.044	0.155	-0.137	0.012	-0.046	-0.020	-0.115	0.637	1.000	0.522	0.204	
	-0.119	0.474	-0.215	-0.195	-0.148	-0.120	-0.049	-0.186	1.000	0.653	0.020	
**9**	0.030	0.416	0.000	-0.208	-0.238	-0.145	-0.247	-0.331	1.000	0.564	0.085	
	-0.236	-0.206	0.080	0.307	0.173	0.009	0.022	1.000	0.637	0.138	0.083	
	0.211	-0.297	0.149	0.190	-0.017	0.115	0.112	1.000	-0.186	-0.053	-0.183	
**8**	-0.074	-0.285	0.008	0.325	0.137	0.176	0.239	1.000	-0.331	-0.348	-0.196	
	0.021	-0.179	0.114	0.056	-0.025	0.239	1.000	0.022	-0.115	-0.271	0.028	
	0.131	-0.291	0.366	0.226	0.009	0.163	1.000	0.112	-0.049	-0.069	-0.065	
**7**	0.044	-0.295	-0.072	0.243	0.343	0.411	1.000	0.239	-0.247	-0.439	0.149	
	0.096	-0.399	0.194	0.096	0.173	1.000	0.239	0.009	-0.020	-0.001	-0.176	
	0.152	-0.491	0.308	0.266	0.397	1.000	0.163	0.115	-0.120	-0.116	-0.040	
**6**	-0.044	-0.386	0.146	0.275	0.365	1.000	0.411	0.176	-0.145	-0.360	0.173	
	-0.386	-0.408	0.398	0.699	1.000	0.173	-0.025	0.173	-0.046	0.042	-0.231	
	-0.213	-0.363	0.311	0.432	1.000	0.397	0.009	-0.017	-0.148	-0.264	-0.236	
**5**	-0.072	-0.350	0.129	0.518	1.000	0.365	0.343	0.137	-0.238	-0.248	-0.090	
	-0.488	-0.353	0.416	1.000	0.699	0.096	0.056	0.307	0.012	0.017	-0.254	
	-0.122	-0.472	0.546	1.000	0.432	0.266	0.226	0.190	-0.195	-0.324	-0.426	
**4**	-0.339	-0.113	0.141	1.000	0.518	0.275	0.243	0.325	-0.208	-0.476	-0.254	
	-0.264	-0.377	1.000	0.416	0.398	0.194	0.114	0.080	-0.137	0.008	-0.228	
	-0.029	-0.526	1.000	0.546	0.311	0.308	0.366	0.149	-0.215	-0.167	-0.364	
**3**	-0.184	-0.164	1.000	0.141	0.129	0.146	-0.072	0.008	0.000	-0.072	-0.169	

	0.044	1.000	-0.377	-0.353	-0.408	-0.399	-0.179	-0.206	0.155	0.086	0.175
	-0.233	1.000	-0.526	-0.472	-0.363	-0.491	-0.291	-0.297	0.474	0.250	0.432
**2**	-0.145	1.000	-0.164	-0.113	-0.350	-0.386	-0.295	-0.285	0.416	0.292	0.233
	1.000	0.044	-0.264	-0.488	-0.386	0.096	0.021	-0.236	-0.044	-0.090	0.117
	1.000	-0.233	-0.029	-0.122	-0.213	0.152	0.131	0.211	-0.119	0.169	-0.092
**1**	1.000	-0.145	-0.184	-0.339	-0.072	-0.044	0.044	-0.074	0.030	0.119	0.023
	**1**	**2**	**3**	**4**	**5**	**6**	**7**	**8**	**9**	**10**	**11**

Correlation matrix for the transcriptome domain plot (Figure 4B)

	0.082	-0.254	-0.325	-0.244	0.583	0.250	0.581	-0.100	-0.298	0.052	1.000	young
	-0.132	-0.316	-0.525	-0.192	0.510	-0.019	0.534	0.011	-0.202	0.319	1.000	middle
**11**	-0.265	-0.204	-0.224	-0.014	0.636	0.206	0.695	0.249	-0.409	0.458	1.000	old
	-0.063	0.051	-0.056	-0.037	-0.012	-0.036	-0.034	0.839	0.767	1.000	0.052	
	-0.022	0.035	-0.160	0.054	0.325	-0.022	0.174	0.168	0.174	1.000	0.319	
**10**	-0.194	0.015	-0.177	0.051	0.410	0.383	0.282	0.322	0.117	1.000	0.458	
	0.015	0.327	0.099	0.102	-0.406	-0.005	-0.355	0.852	1.000	0.767	-0.298	
	-0.104	0.073	0.266	0.116	-0.519	0.056	-0.411	0.352	1.000	0.174	-0.202	
**9**	0.288	0.234	-0.234	-0.183	-0.504	-0.151	-0.532	0.263	1.000	0.117	-0.409	
	-0.069	0.124	0.116	0.015	-0.185	-0.051	-0.149	1.000	0.852	0.839	-0.100	
	0.061	0.106	0.199	0.009	-0.095	-0.078	-0.256	1.000	0.352	0.168	0.011	
**8**	-0.260	-0.172	-0.113	-0.062	0.147	0.177	0.033	1.000	0.263	0.322	0.249	
	0.072	-0.457	-0.429	-0.440	0.614	0.505	1.000	-0.149	-0.355	-0.034	0.581	
	-0.209	-0.283	-0.613	-0.150	0.648	0.194	1.000	-0.256	-0.411	0.174	0.534	
**7**	-0.312	-0.300	-0.222	-0.013	0.692	0.303	1.000	0.033	-0.532	0.282	0.695	
	0.179	0.204	0.136	-0.107	0.372	1.000	0.505	-0.051	-0.005	-0.036	0.250	
	0.047	0.306	0.185	0.001	0.152	1.000	0.194	-0.078	0.056	-0.022	-0.019	
**6**	-0.153	0.123	0.216	0.263	0.500	1.000	0.303	0.177	-0.151	0.383	0.206	
	0.092	0.009	-0.147	0.065	1.000	0.372	0.614	-0.185	-0.406	-0.012	0.583	
	0.033	0.120	-0.348	0.159	1.000	0.152	0.648	-0.095	-0.519	0.325	0.510	
**5**	-0.289	0.112	0.098	0.346	1.000	0.500	0.692	0.147	-0.504	0.410	0.636	

	-0.037	0.710	0.454	1.000	0.065	-0.107	-0.440	0.015	0.102	-0.037	-0.244
	0.017	0.572	0.257	1.000	0.159	0.001	-0.150	0.009	0.116	0.054	-0.192
**4**	-0.139	0.600	0.392	1.000	0.346	0.263	-0.013	-0.062	-0.183	0.051	-0.014
	-0.037	0.555	1.000	0.454	-0.147	0.136	-0.429	0.116	0.099	-0.056	-0.325
	0.261	0.493	1.000	0.257	-0.348	0.185	-0.613	0.199	0.266	-0.160	-0.525
**3**	0.101	0.392	1.000	0.392	0.098	0.216	-0.222	-0.113	-0.234	-0.177	-0.224
	0.046	1.000	0.555	0.710	0.009	0.204	-0.457	0.124	0.327	0.051	-0.254
	0.439	1.000	0.493	0.572	0.120	0.306	-0.283	0.106	0.073	0.035	-0.316
**2**	0.428	1.000	0.392	0.600	0.112	0.123	-0.300	-0.172	0.234	0.015	-0.204
	1.000	0.046	-0.037	-0.037	0.092	0.179	0.072	-0.069	0.015	-0.063	0.082
	1.000	0.439	0.261	0.017	0.033	0.047	-0.209	0.061	-0.104	-0.022	-0.132
**1**	1.000	0.428	0.101	-0.139	-0.289	-0.153	-0.312	-0.260	0.288	-0.194	-0.265
	**1**	**2**	**3**	**4**	**5**	**6**	**7**	**8**	**9**	**10**	**11**
